# Role of the Wnt-β-catenin signaling pathway in melanoma-mediated dendritic cell tolerization

**DOI:** 10.1186/2051-1426-1-S1-P153

**Published:** 2013-11-07

**Authors:** Alisha Holtzhausen, Kathy Evans, Brent A Hanks

**Affiliations:** 1 Internal Medicine, Medical Oncology, Duke University Medical Center, Durham, NC, USA

## 

Recent studies have shown that tumor immune evasion mechanisms significantly contribute to immunotherapy failure. Emerging data is indicating that tumor-associated dendritic cells (DCs) undergo phenotypic tolerization and promote the differentiation and activation of regulatory T cell (Treg) populations to generate local immune subversion. The critical role of DCs in orchestrating anti-tumor immunity suggests that the signaling pathways that regulate DC tolerogenesis may be promising targets for pharmacologically augmenting immunotherapy efficacy. Previous work has shown that the β-catenin signaling pathway plays a potential role in the DC tolerization process. Others have demonstrated that the DC-expressed enzyme, indoleamine 2,3-dioxygenase (IDO), promotes tumor-induced immune tolerance by catalyzing the conversion of the amino acid tryptophan into kynurenine. Other investigators have demonstrated melanomas to upregulate the expression of several Wnt ligands during their malignant transformation. Using a transgenic murine model of melanoma, we have determined that melanoma-associated DCs exhibit elevated levels of expression of IDO and other β-catenin target genes and demonstrate diminished T cell stimulatory capacity in vitro. Further studies have shown that Wnt3a and Wnt5a induce the expression and enzymatic activity of IDO in myeloid DCs (mDCs) in a β-catenin-dependent fashion while IDO reporter assays suggest this process to be mediated by direct promoter activation. This data suggest the Wnt-β-catenin signaling pathway to be a potential target for suppressing melanoma-induced DC tolerization and enhancing anti-tumor immunity. Indeed, both small molecule inhibition and genetic silencing of the PORCN acyl transferase enzyme which is necessary for Wnt secretion, abrogated B16 melanoma paracrine induction of β-catenin signaling and IDO expression by local mDCs. Further studies have shown PORCN-silenced B16 melanomas to exhibit reduced tumorigenecity in vivo and that this effect correlated with decreased mDC IDO expression and reduced levels of local Tregs. We therefore hypothesize that melanoma-expressed Wnt ligands induce DC tolerogenesis and stimulate β-catenin-dependent IDO expression and subsequent Treg generation in the tumor microenvironment, resulting in the development of an immunotolerant state and ultimately allowing for disease progression. Further, we propose that the pharmacological inhibition of this pathway can reverse this mechanism of immune evasion and enhance other immunotherapeutic approaches. Future work will include testing the ability of the small molecule PORCN inhibitor to stimulate the generation of tumor antigen-specific T cell responses in an autochthonous transgenic melanoma model.

